# Influencial factors of the performance of interferon-γ release assays in the diagnosis of childhood tuberculosis

**DOI:** 10.1007/s10238-014-0296-3

**Published:** 2014-06-13

**Authors:** Tao Li, Lei Bao, Ni Diao, Feng Sun, Yan Gao, Ka-Wing Wong, Xiuhong Xi, Xuhui Liu, Sen Wang, Jing Wu, Ma Hui, Xiaoyong Fan, Ying Zhang, Wenhong Zhang, Shuihua Lu

**Affiliations:** 1 Shanghai Public Health Clinical Center, Fudan University, Shanghai, China; 2 Department of Infectious Diseases, Huashan Hospital, Fudan University, Shanghai, China; 3 School of Basic Medical Sciences, Shanghai Medical College, Fudan University, Shanghai, China; 4 Department of Molecular Microbiology and Immunology, Bloomberg School of Public Health, Johns Hopkins University, Baltimore, MD USA; 5 Institutes of Biomedical Sciences, Fudan University, Shanghai, China

**Keywords:** Interferon-γ release assays, Childhood tuberculosis, Diagnosis, Prednisolone, Age

## Abstract

Diagnosis of active tuberculosis (TB) in children remains difficult. This study aimed at evaluating the ability of interferon-gamma release assays (IGRAs) in the detection of active TB in human immunodeficiency virus-negative children vaccinated with Bacille Calmette–Guérin and investigating the effect of prednisolone treatment on the IGRAs performance. Among the 162 children with suspected TB disease recruited in China, 60 were tested with QuantiFERON-TB Gold In Tube (QFT-GIT) and 102 were tested with T-SPOT.TB. QFT-GIT presented a sensitivity of 83.9 % (95 % CI 66.9–93.4 %) and a specificity of 88.5 % (95 % CI 70.2–96.8 %), while T-SPOT.TB had a sensitivity of 75.9 % (95 % CI 63.4–85.2 %) and a specificity of 94.7 % (95 % CI 81.8–99.5 %). The positive predictive value was high in both assays, 92.9 % for QFT-GIT and 95.7 % for T-SPOT.TB. In total of these two kinds of IGRAs, false negative rate was significantly higher in children receiving systemic prednisolone (1 mg/kg/day) therapy for >1 week (two tested with T-SPOT.TB and five tested with QFT-GIT) than in those with ≤1 week of prednisolone therapy and without prednisolone therapy (57.1 vs. 18.3 %, *p* = 0.035). There was no significant difference of the positive rate of both tests in children <5 years old compared with those ≥5 years old. Both types of IGRAs showed good diagnostic values in detecting childhood TB before microbiological evidence was available. Glucocorticoids had a significant negative influence on IGRAs if treated for >1 week. Age made no difference on the performance of these tests in children.

## Introduction

Tuberculosis (TB), caused by *Mycobacterium tuberculosis* (MTB), has infected approximately one third of the global population. There were 8.7 million incident cases and 1.42 million deaths in 2011. Data on the global burden of childhood TB was not available until 2012, when the World Health Organization (WHO) first reported estimates of 490,000 new cases and 64,000 deaths in 2011 [1]. Although China ranks second among the high-burden countries for tuberculosis, with a total of 1.4 million TB patients with 1 million new cases per year, there is no epidemiological data of childhood TB in China.

Diagnosis of childhood tuberculosis is especially difficult because of the atypical clinical and radiological presentations and lack of sputum production in young children. Tuberculin skin test (TST) has been the main screening test for MTB infection since last century. However, false-positive results could occur because diagnostic antigens used in TST have cross-reactions with non-tuberculous mycobacteria or Bacille Calmette–Guérin (BCG) vaccine [2]. T cell-based IFN-γ release assays (IGRAs) measure the release of IFN-γ after in vitro stimulation with MTB-specific antigens, such as early secreted antigenic target 6 (ESAT-6) and culture filtrate protein 10 (CFP 10). Thus, the IGRAs have the ability to reduce false-positive results and show higher sensitivity and specificity than TST [3–5]. There are two commercially available IGRAs tests: the whole blood-based QuantiFERON-TB Gold in Tube (QFT-GIT) and the PBMC-based T-SPOT.TB.

The diagnostic value of IGRAs has focused much less on children than adults [6]. A study conducted in Germany, a country which abandoned BCG vaccination since 1998, found that the sensitivity of QFT-GIT for the diagnosis of childhood TB disease was 93 % [7]. A prospective study of 210 children in Houston, Texas showed that the sensitivity of T-SPOT.TB in culture-confirmed tuberculosis children was 92 % [8]. However, data were sparse in countries with a high coverage of BCG vaccination and a high incidence of TB. In this study, we evaluated the utility of QFT-GIT and T-SPOT.TB among BCG-vaccinated and HIV-negative children for diagnosis of TB for the first time in China. We also investigated the influence of immunosuppressive treatment such as glucocorticoids on the diagnostic performance of IGRA tests.

## Methods

### Study setting and population

Children clinically suspected for active tuberculosis with symptoms such as ① fever, night sweat or weight loss, ② cough, expectoration, chest pain or hemoptysis, ③ headache, fever or unconsciousness, ④ rash, subcutaneous mass or lymphadenectasis hospitalized at Shanghai Public Health Clinical Center of Fudan University on suspicion of tuberculosis disease were recruited after informed consent was obtained from guardians. All the children were tested with either QFT-GIT or T-SPOT.TB after enrollment. None of the recruited children were co-infected with HIV.

Based on the clinical, radiological, pathological and microbiological findings collected at baseline and during follow-up, the children were grouped into four different diagnostic categories. Definition of case categories is listed in Table 1 [9]. Follow-up visits were scheduled at 1, 2, 5, 6 and 12 months after enrollment. Patients were asked to make unscheduled visits in case their clinical conditions got worse. Results of the QFT-GIT and T-SPOT.TB had no effect on treatment decisions. The study was approved by the institutional review board of Shanghai Public Health Clinical Center.

**Table 1:** Diagnostic classification of patients

Clinical diagnostic groups	Definition of case categories
Confirmed tuberculosis	Children with clinical specimens positive for *M. tuberculosis* in culture or acid-fast bacilli in microscopy or PCR
Highly probable tuberculosis	One or more of the following symptoms 1. Chest X-ray highly suggestive of active tuberculosis Miliary findings in HIV-negative children Nonpyogenic pleural effusion Cavitation associated with subacute or chronic pneumonia Hila or mediastinal lymphadenopathy 2. Histology or biopsy tissue showing caseating granuloma 3. Abdominal mass or ascites with abdomina lymphadenopathy on ultrasound scan 4. Spinal gibbus, destruction or compression fracture of vertebral bodies or paravertebral abscess 5. Symptoms of meningitis with tests of CSF consistent with tuberculosis meningitis 6. Clinical improvement with anti-tuberculosis treatment.
Possible tuberculosis	Children who do not fulfill criteria for confirmed or highly probable tuberculosis, but still could not be excluded for active tuberculosis
Not tuberculosis	Excluded from active tuberculosis with an established alternative diagnosis, and sustained recovery during 12 months of follow-up

### T-SPOT.TB^®^ and QuantiFERON-TB gold in tube^®^

The two types of IGRAs tests were performed according to the manufacturers’ instructions.

T-SPOT.TB (Oxford Immunotec Ltd., Oxford, UK) is an ELISPOT assay to detect IFN-γ induced by ESAT-6 and CFP-10. The spot forming cells (SFCs) were counted using an automated ELISPOT Reader (AID systems, Strasberg, Germany). Test result of T-SPOT.TB assay would be considered positive if (1) Panel A (containing peptide antigens derived from ESAT-6) and/or Panel B (containing peptide antigens derived from CFP-10) had six or more spots than the negative control when the spots of the negative control ≤5; (2) the number of spots in Panel A or B was at least two times higher than that of the negative control when there were more than five spots for the negative control. Results were checked by other lab workers and, if necessary, corrected by manual counting.

QuantiFERON-TB Gold in Tube (Cellestis Limited, Carnegie, Victoria, Australia) was performed in two stages. First, 1 mL of whole blood was collected into each of the specialized blood collection tubes, which included a nil control tube, a TB antigen tube(containing ESAT-6, CFP-10 and TB7.7), and a mitogen tube as a positive control. The tubes were transferred to a 37 °C incubator. After 16–24 h of incubation, the tubes were centrifuged and the supernatant was collected. Then the amount of IFN-γ (IU/mL) in the supernatant was measured by ELISA. IFN-γ secreted specific to TB antigen was calculated by subtracting the level in nil tube from that in TB antigen tube. Test result would be considered positive if the value is ≥0.35 IU/mL and ≥25 % more than the nil control with any value for mitogen tube, and negative if the concentration of the positive control minus negative control is ≥0.5 IU/ml, with the calculated value ≥0.35 IU/mL but <25 % more than the nil control value, or <0.35 IU/ml. Results were interpreted as indeterminate if ① the calculated value was <0.35, or ≥0.35 IU/mL but <25 % more than the nil control, both with the positive control minus negative control <0.5 IU/mL; ② the value of the nil tube was >8.0 IU/mL.

### Statistical analysis

Cases with indeterminate result were included into the sum of cases in the calculation of sensitivity and specificity, but not into positive predictive value (PPV) and negative predictive value (NPV). Positive rate in different groups were compared using the *χ* ^2^ test. 95 % confidence intervals (CIs) were calculated using the Wilson score method. *p*<0.05 were considered to be statistically significant. Data were analyzed by GraphPad Prism 5.

## Results

### Study population

**Fig. 1:**
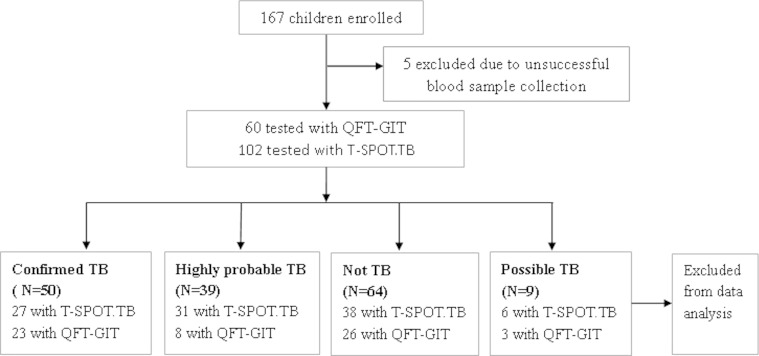
Recruitment and diagnostic classification of all participants

Of the 167 children enrolled in this study, five were excluded due to unsuccessful blood collection, 102 were tested with T-SPOT.TB and 60 with QFT-GIT. None of these children had been tested both with QFT-GIT and T-SPOT.TB. Among the 102 children tested with T-SPOT.TB, 27 were diagnosed with confirmed tuberculosis, 31 with highly probable tuberculosis, and 38 without tuberculosis. Among the 60 children with QFT-GIT test, 23 were classified as confirmed tuberculosis, eight with highly probable tuberculosis, and 26 without tuberculosis. Nine cases (six tested with T-SPOT.TB and three tested with QFT-GIT) were unable to get a definite diagnosis and were excluded from data analysis (Fig. 1). For children diagnosed with highly probable TB, hila or mediastinal lymphadenopathy was the most common symptoms. All children with confirmed and highly probable TB received anti-tuberculosis treatment. One child with confirmed TB was lost for follow-up. The remaining 88 children with confirmed TB or highly probable TB showed sustained clinical improvement after initiation of anti-tuberculosis therapy. All the 64 children who were not TB had no active tuberculosis during 12-month follow-up. Clinical samples for microbiological identification included gastric aspirates, induced sputum samples, bronchoscopy lavage fluid, samples from lymph node aspiration, samples from percutaneous biopsy of pulmonary under CT guidance, pericardial stripping organization, pleural fluid samples, ascites, bone marrow and cerebrospinal fluid (CSF). The main characteristics are listed in Table 2.

**Table 2:** Demographic and clinical details of study subjects

Diagnostic classification						
Characteristic	Confirmed tuberculosis (*N* = 50)		Highly probable tuberculosis (*N* = 39)		Not tuberculosis (*N* = 64)	
	T-SPOT.TB (*N* = 27) no. %	QFT-GIT (*N* = 23) no. %	T-SPOT.TB (*N* = 31) no. %	QFT-GIT (*N* = 8) no. %	T-SPOT.TB (*N* = 38) no. %	QFT-GIT (*N* = 26) no. %
Age, median months (IQR)	45 (2–156)	56 (3–168)	40 (3–160)	42 (4–179)	52 (2–160)	57.2 (3–156)
Male sex	15 (55.6)	12 (52.2)	15 (48.4)	4 (50.0)	21 (55.3)	13 (50.0)
BCG vaccinated	27 (100.0)	23 (100.0)	31 (100.0)	8 (100.0)	38 (100.0)	26 (100.0)
Fever	14 (51.9)	14 (60.9)	16 (51.6)	4 (50.0)	14 (36.8)	19 (73.1)
Night sweats	23 (85.2)	20 (87.0)	11 (35.5)	6 (75.0)	21 (55.3)	15 (57.7)
Cough >2 weeks	19(70.1)	11 (47.8)	23 (74.2)	5 (62.5)	5 (13.2)	3 (11.5)
Chest radiogragh						
Normal	3 (8.1)	0 (0.0)	4 (12.9)	2 (25.0)	27 (71.1)	8 (30.8)
Abnormal^†^	23 (85.2)	23 (100.0)	27 (87.1)	6 (75.0)	8 (21.1)	16 (61.5)
Not done/unavailable	0 (0.0)	0 (0.0)	0 (0.0)	0 (0.0)	3 (7.9)	2 (7.7)
Tuberculosis type						
PTB only	5 (18.5)	5 (21.7)	8 (25.8)	4 (50.0)	0 (0.0)	0 (0.0)
EPTB only	6 (22.2)	2 (8.7)	4 (12.9)	3 (37.5)	0 (0.0)	0 (0.0)
Both PTB and EPTB	15 (55.6)	16 (69.6)	19 (61.3)	1 (12.5)	0 (0.0)	0 (0.0)
HIV-infected	0 (0.0)	0 (0.0)	0 (0.0)	0 (0.0)	0 (0.0)	0 (0.0)
Receive prednisolone (1 mg/kg/day >1 week)	1 (3.7)	4 (17.4)	1 (3.2)	1 (12.5)	0 (0.0)	0 (0.0)
Receive prednisolone (1 mg/kg/day ≤1 week)	2 (7.4)	7 (30.4)	2 (6.5)	0 (0.0)	0 (0.0)	0 (0.0)
History of tuberculosis close contact	2 (7.4)	5 (21.7)	1 (3.2)	2 (25.0)	0 (0.0)	0 (0.0)

*PTB* pulmonary tuberculosis, *EPTB* extra-pulmonary tuberculosis

^†^Abnormal chest radiograph included miliary findings, tuberculoma, pleural effusion, cavitation, lymphadenectasis and some other abnormal findings

### Results of T-SPOT.TB and QFT-GIT assay

After excluding those with possible TB, results of the remaining 153 children are listed in Table 3. The positive rate of T-SPOT.TB was 74.1 % (20/27) in children with confirmed TB and 77.4 % (24/31) in children with highly probable TB, while the positive rate of QFT-GIT was 87.0 % (20/23) in children with confirmed TB and 75.0 % (6/8) in children with highly probable TB. Among children with other diseases than TB, 2 were positive for T-SPOT.TB, 2 positive for QFT-GIT, and one child got an indeterminate result of QFT-GIT.

**Table 3:** Results of T-SPOT.TB and QFT-GIT in each diagnostic group

Result of IGRA	Confirmed TB		Highly probable TB		Not TB	
	T-SPOT.TB (*N* = 27) no. %	QFT-GIT (*N* = 23) no. %	T-SPOT.TB (*N* = 31) no. %	QFT-GIT (*N* = 8) no. %	T-SPOT.TB (*N* = 38) no. %	QFT-GIT (*N* = 26) no. %
Positive	20 (74.1)	20 (87.0)	24 (77.4)	6 (75.0)	2 (5.3)	2 (7.7)
Negative	7 (25.9)	3 (13.0)	7 (22.6)	2 (25.0)	36 (94.7)	23 (88.5)
Indeterminate	–	0 (0.0)	–	0 (0.0)	–	1 (3.8)

### Comparison of the sensitivity and specificity of the T-SPOT.TB and QFT-GIT

The sensitivity of T-SPOT.TB and QFT-GIT for the diagnosis of active TB was 75.9 % (63.4–85.2 %) and 83.9 % (95 % CI 66.9–93.4 %), respectively. The specificity of T-SPOT.TB and QFT-GIT was 94.7 % (95 % CI 81.8–99.5 %) and 88.5 % (95 % CI 78.6–99.2 %), respectively. The PPV of T-SPOT.TB and QFT-GIT was similar (95.7 vs. 92.9 %), and NPV of T-SPOT.TB was about 10 % higher than QFT-GIT (82.1 vs. 72.0 %) (Table 4).

**Table 4:** Evaluation index of T-SPOT.TB and QFT-GIT

	Sensitivity (%, 95 % CI)	Specificity (%, 95 % CI)	PPV (%, 95 % CI)	NPV (%, 95 % CI)	Positive LR	Negative LR
T-SPOT.TB	75.9 (63.4–85.2)	94.7 (81.8–99.5)	95.7 (84.7–99.6)	72.0 (58.2–82.6)	14.3	0.25
QFT-GIT	83.9 (66.9–93.4)	88.5 (70.2–96.8)	92.9 (76.3–99.1)	82.1 (63.9–92.6)	7.3	0.18

*PPV* positive predictive value, *NPV* negative predictive value, *LR* likelihood ratio

### The effect of prednisolone treatment on the risk of false negative results of T-SPOT.TB and QFT-GIT

**Fig. 2:**
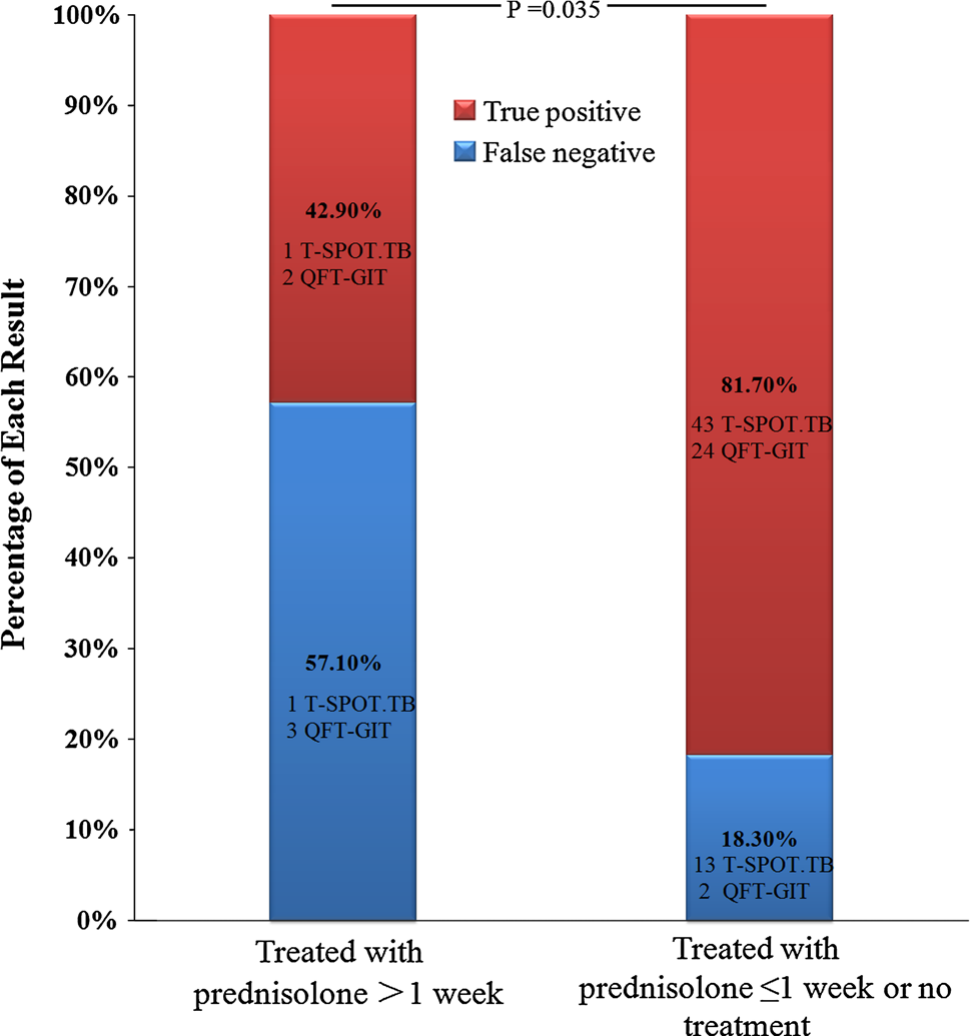
Effects of prednisolone treatment on T-SPOT.TB and QFT-GIT results of children with ATB (*w* week)

Six children tested with T-SPOT.TB and 12 children tested with QFT-GIT had received treatment with prednisolone in our study. Prednisolone was prescribed to control the inflammatory reaction in children with tuberculous pleuritis or tuberculous meningitis. In comparison with the numbers of SFCs in panel A and B in T-SPOT.TB or the concentration of IFN-γ in TB antigen tube in QFT-GIT, a tendency of decrease in IFN-γ release after prednisolone therapy was both observed in T-SPOT.TB (mean SFCs for panel A: 28.8 vs. 58.2, mean SFCs for panel B: 34 vs. 68.9) and QFT-GIT (mean concentration: 3.98 IU/ml vs. 5.35 IU/ml), but the difference was not significant (*p* > 0.05).According to the duration of prednisolone therapy prior to IGRAs, we regrouped the cases into children with prednisolone (1 mg/kg/day) for >1 week and with prednisolone (1 mg/kg/day) for ≤1 week or without prednisolone treatment. Two children tested with T-SPOT.TB and five children tested with QFT-GIT had been treated with prednisolone for more than 1 week. The false negative rate of T-SPOT.TB and QFT-GIT was 57.1 % (one tested with T-SPOT.TB and three with QFT-GIT) in children with >1 week of prednisolone therapy higher than 18.3 % (13 tested with T-SPOT.TB and two with QFT-GIT) of others with shorter/no therapy (*p* = 0.035) (Fig. 2).

### The effect of age on the results of T-SPOT.TB and QFT-GIT

We stratified children into two groups according to their age: children <5 years old and children ≥5 years old. For children with confirmed and highly probable TB, positive rates of T-SPOT.TB was 66.7 % among children <5 years old and 21.3 % higher (88.0 %) among children ≥5 years old. On the contrary, the positive rate of QFT-GIT was 90 % in children <5 years old which was 17.3 % higher than children ≥5 years old. However, the difference was of no significance (*p* > 0.05). For children with not TB, positive rates were a little higher in elder children than in younger children (16.7 vs. 3.1 % for T-SPOT.TB and 10.0 vs. 6.3 % for QFT-GIT), but the differences were also not significant (*p* > 0.05).

## Discussion

Commonly used methods for the diagnosis of childhood tuberculosis have their shortcomings. TST has limited diagnostic value in the areas with a high coverage rate of BCG vaccination because of cross-reactions. Meanwhile, positive rate for sputum culture was low, even for those who got positive result, 50 % were negative for fast sputum-smear test [10]. Therefore, new methods with high accuracy and convenience are urgently needed for better diagnosis of childhood TB. T cell-based IFN-γ release assays (IGRAs) are based on the host immune response against *M. tuberculosis* specific antigens, which gives them higher specificity than TST. Previous studies have shown that both QFT-GIT and T-SPOT.TB presented high sensitivity and specificity in adults [5, 11, 12], and showed good value in diagnosing active tuberculosis, and identifying latent TB infection [11, 13, 14]. However, there are disagreements on the value of IGRAs in the diagnosis of childhood tuberculosis [7, 15]. Some studies showed that IGRAs had no obvious advantages in children [16–18].

Based on our data, the sensitivity and specificity of QFT-GIT for the diagnosis of childhood TB were both higher than 80 % (83.9 and 88.5 %), while T-SPOT.TB seemed to have a lower sensitivity (75.9 %) and a higher specificity (94.7 %) than QFT-GIT without statistical difference. In a meta-analysis done by Sollai S et al.[19] , sensitivity of both QFT-GIT and T-SPOT.TB was about 10 % higher among children with microbiologically confirmed TB than that in all TB children. In our study, the rate of bacteriologically confirmed TB was 38.3 % (23/60) for QFT-GIT and 26.5 % (27/102) for T-SPOT.TB. The difference of the rate between children receiving two IGRAs may lead to the trend of higher sensitivity of QFT-GIT than T-SPOT.TB. Both types of IGRAs had a high PPV, 95.7 % for T-SPOT.TB and 92.9 % for QFT-GIT. IGRAs presented a better diagnostic value in children in our study than the previous study in adults [20]. Prevalence of latent infection with MTB increases with age, IGRAs performed among children are less affected by latent infection, which increases its value for detecting active tuberculosis in children. For children with suspected TB in a high prevalence area, the high PPV and specificity of the IGRAs would facilitate ruling in childhood TB when a positive result is provided and anti-tuberculosis therapy would accordingly start on those child patients much earlier than microbiological evidence is available. However, we should pay attention that since IGRAs cannot differentiate active TB from latent TB infection, either test should not be used alone to confirm or rule out active TB.

Approximately 20.2 % (18/89) of all children with confirmed and highly probable tuberculosis were treated with prednisolone before tested with IGRAs. A statistical difference in the false negative rate was shown between children treated for >1 week and those treated with shorter or no prednisolone therapy (57.1 vs. 18.3 %, *p* = 0.035). Prednisolone can broadly influence biochemical behavior of most cells, and its anti-inflammatory and immunosuppressive action may impair the T cell proliferation and differentiation in response to TB-specific antigens as shown by the weakened ability of T cells to secrete IFN-γ in IGRAs. Previous study showed that immunocompromised children including those receiving glucocorticoid treatment had a higher risk of indeterminate result for QFT-GIT test [21], and glucocorticoids taken by oral route impair the response to mitogen more than taken by other ways [22]. In our study, all children took prednisolone intravenously and none of these 12 children got an indeterminate result in QFT-GIT. Young age was considered to influence the performance of IGRAs in children, especially for QFT-GIT. Studies have shown that for children aged <4 years old, frequency of indeterminate results was higher [15, 23–26]. However, only one indeterminate result was detected in our study. Studies for the usage of IGRAs in young children <5 years of age were limited [27, 28], in our study, positive rates of both IGRAs and QFT-GIT were not significantly different between children <5 years old and ≥5 years old.

Our conclusion was limited by the small number of subjects included in the study. A large scale study of effects of glucocorticoids with different duration on performance of IGRAs and the factors associated with indeterminate results in QFT-GIT is needed in the future.

In summary, both IGRA tests showed a good diagnostic value in detecting active childhood tuberculosis in children suspected for active TB in a TB epidemic country. Low incidence of latent TB infection makes IGRAs a better tool in children than in adults. In addition, glucocorticoids can have a negative influence on IGRAs if treated for >1 week. In children, age did not affect the performance of IGRAs.
